# Prognostic value of the pretreatment neutrophil-to-lymphocyte ratio in cervical cancer: a meta-analysis and systematic review

**DOI:** 10.18632/oncotarget.14541

**Published:** 2017-01-06

**Authors:** Jiayuan Wu, Manyu Chen, Caixia Liang, Wenmei Su

**Affiliations:** ^1^ Nutritional Department, The Affiliated Hospital of Guangdong Medical University, Zhanjiang 524001, China; ^2^ Department of Oncology, The Affiliated Hospital of Guangdong Medical University, Zhanjiang 524001, China

**Keywords:** neutrophil-to-lymphocyte ratio, cervical cancer, prognosis, meta-analysis

## Abstract

The prognostic value of pretreatment neutrophil-to-lymphocyte ratio (NLR) in cervical cancer remains controversial. We conducted a meta-analysis based on the data from 13 studies with 3729 patients to evaluate the association between the pretreatment NLR and the clinical outcomes of overall survival and progression-free survival in patients with cervical cancer. The relationship between NLR and clinicopathological parameters was also assessed. Hazard ratio (HR) or odds ratio (OR) with its 95% confidence interval (CI) was used as the effect size estimate. Our analysis indicated that elevated pretreatment NLR was a poor prognostic marker for patients with cervical cancer because it predicted unfavorable overall survival (HR = 1.375, 95% CI: 1.200–1.576) and progression-free survival (HR = 1.646, 95% CI: 1.313–2.065). Increased NLR is also significantly associated with the larger tumor size (OR = 1.780, 95% CI: 1.090–2.908), advanced clinical stage (OR = 2.443, 95% CI: 1.730–3.451), and positive lymph node metastasis (OR = 2.380, 95% CI: 1.775–3.190). By these results, high pretreatment NLR predicted a shorter survival period for patients with cervical cancer, and it could be served as a novel index of prognostic evaluation in patients with cervical cancer.

## INTRODUCTION

Cervical cancer (CC) is the second most common type of gynecologic cancer worldwide, leading approximately 500,000 new diagnosed cases and 275,000 deaths annually [1]. Nearly one-third of CC patients die from disease recurrence or progression [2]. Up to now, the International Federation of Obstetricians and Gynecologists (FIGO) tumor staging system, lymph node status, tumor size, histological grade, and depth of invasion were well known to be the prognostic factors of patients with CC [3–5]. Except the FIGO stage, other variables can only be evaluated after surgery. However, clinical staging has been shown to be frequently inaccurate in predicting the prognosis of CC patients, especially in some patients with advanced disease [6]. Therefore, a pretreatment and effective parameter to evaluate survival probability and prognosis of CC is necessary for decision-making concerning clinical therapy.

It is well-known that many cancers develop from sites of infection, chronic irritation, and inflammation. Inflammation influences each single step of tumorigenesis, from tumour initiation to promotion and metastatic progression [7]. Accumulating evidence shows that inflammatory cells in the tumour microenvironment plays a critical role in tumor development through inducing proliferation and survival of cancer cells, promoting angiogenesis and metastasis, suppressing the adaptive immune system, and altering the response to hormones and chemotherapeutic agents [8]. Pre-therapeutic indices of systemic inflammation, such as C-reactive protein (CRP) [9], neutrophil-to-lymphocyte ratio (NLR), platelet-to-lymphocyte ratio [10], and modified Glasgow prognostic score (mGPS) [11], have been investigated to provide prognostic information for CC.

Among these inflammatory markers, NLR, represented as a combination of circulating neutrophils and lymphocytes counts, has been gained notable interest particularly. Neutrophils and lymphocytes are the principal components of the tumor-related stroma, and are closely correlated with local inflammation and immune responses, respectively [12]. NLR indicates the balance of the inflammatiory and immune systems, and also reflects the balance between pro-tumor and anti-tumor status, making it a useful index for predicting prongisis in malignance [13]. NLR has been reported to be associated with worse prognosis in patients with many cancer types, including lung cancer [14, 15], colorectal cancer [16, 17], gastric cancer [18, 19], esophageal cancer [20, 21], hepatocellular cancer [22], pancreatic cancer [23], and renal cell carcinoma [24]. Recent studies have declared the prognostic significance of NLR in patients with CC; however, these studies presented conflicting data due to the variance in study desigen, sample size and patient characteristics. Considering that meta-analyses are useful to integrate results from independent studies for a specified outcome, we conducted a meta-analysis to comprehensively evaluate the prognostic value of NLR in CC patients.

## RESULTS

### Literature search and included studies

**Figure 1:**
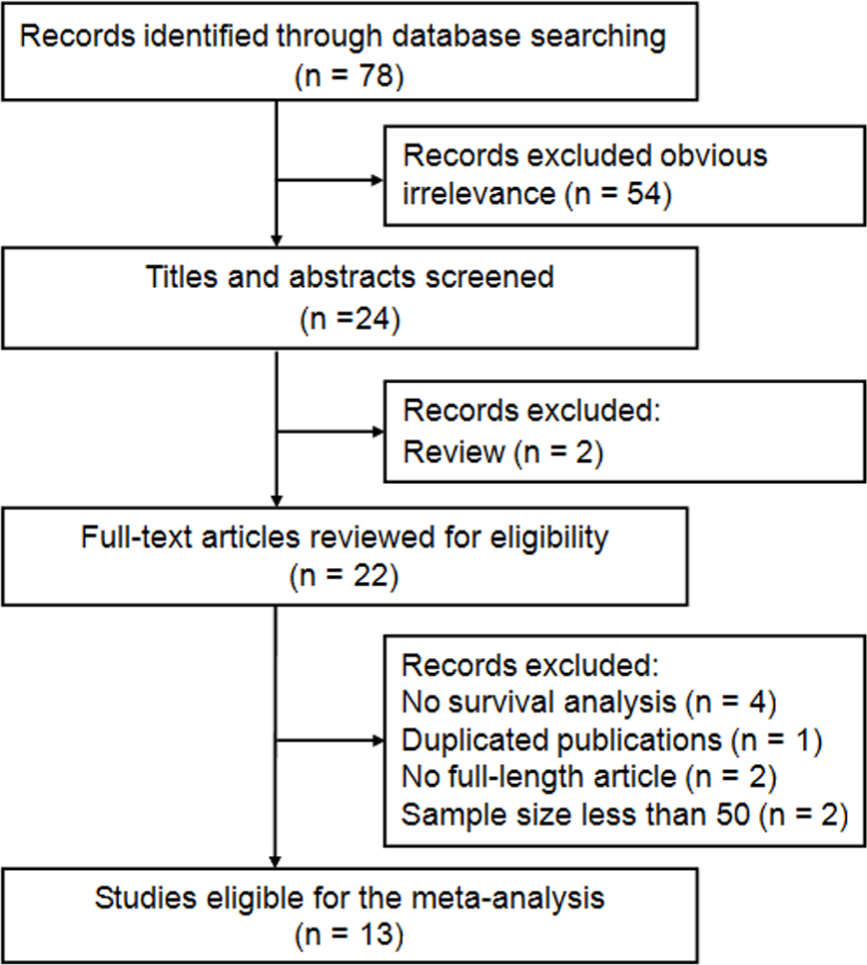
Flow diagram of the study selection process and specific reasons for exclusion in the meta-analysis 78 studies were preretrieved in accordance with the established search strategies. Of these articles, 54 were excluded because of clear lack of relevance. The remaining 24 studies were further screened out through browsing the titles and abstracts, and then 2 were removed based on the eligible criteria. After reading the full texts of 22 studies, 13 eligible studies were finally included in this meta-analysis.

The process of literature search was shown in Figure 1. Initially, 78 papers were generated in the primary electronic search in the major databases. According to the inclusion criteria, 13 full-text articles [25–37] published from 2012 to 2016 were selected for our meta-analysis finally. The main characteristics of the included studies were listed in Table 1. A total of 3729 patients were included. These studies came from China [25, 27, 29, 30, 32–36], Korea [37], Japan [26, 31], and Turkey [28], respectively. Twelve articles reported the outcomes of overall survival (OS) [25–33, 35–37], and 10 studies presented progression-free survival (PFS) as primary outcome [26–28, 31–37]. The hazard ratios (HRs) and the corresponding 95% confidence intervals (CIs) were obtained by multivariate analyses in 8 studies and through univariate or Kaplan–Meier curves in 5 studies [25–27, 34, 36]. Five studies included CC patients with all disease stages (Stages I–IV) [26, 28, 31, 33, 37], seven studies recruited patients with Stages I–II [25, 29, 30, 32, 34–36] and only one study reported the data of CC patients with Stages II–III [27]. The primary treatments were extremely various among the 13 included studies, including concurrent chemoradiotherapy (CCRT) [26–28], surgery with neoadjuvant chemotherapy (NACT) [30, 36], surgery with adjuvant therapy (AT) [25, 29, 32, 35], surgery alone [34], radiotherapy or CCRT [31, 33], and mixed treatments [37]. According to the quality criteria, all cohort studies were of high quality and had scores of six or more.

**Table 1:** Main characteristics of the included studies

Study (year)	Country	Duration	Follow up (months)	Sample	Age (years)	Stage	No. of SCC (%)	Primary treatment	Cut-off value	No. of elevated NLR (%)	Survival outcome	Analysis	Quality
Zheng RR (2016)	China	2005–2012	Median 62.3	795	Median 49.5	IA–IIA	NR	Surgery + AT	2.77	433 (54.5)	OS	Univariate	7
Haraga L (2016)	Japan	2009–2013	NR	95	Median 61.5	IB–IIA	86 (90.5)	CCRT	2.78	50 (52.7)	OS, PFS	Univariate	7
Wang YY (2016)	China	2009–2010	Up to 2015.6	60	Median 53	II–III	NR	CCRT	2.00	36 (60.0)	OS, PFS	Univariate	6
Onal Cem (2016)	Turkey	2006–2014	Median 31.7 (3.7–114.2)	235	Median 57	IB–IVA	218 (92.8)	CCRT	3.03	117 (49.8)	OS, PFS	Multivariate	8
Chen L (2016)	China	2006–2009	Up to 2014.12	407	Median 44	IB–IIA	357 (87.7)	Surgery + AT	2.41	264 (64.9)	OS	Multivariate	8
Zhou WY (2016)	China	2010	NR	75	Median 45	IB–IIB	NR	Surgery + NACT	2.00	39 (52.0)	OS	Multivariate	6
Mizunuma M (2015)	Japan	2005–2013	NR	56	Median 65.1	IB–IV	56 (100)	Radiotherapy or CCRT	2.50	35 (62.5)	OS, PFS	Multivariate	7
Yang WJ (2015)	China	2007–2009	Up to 2014.1	76	Mean 53.28	I–IVA	56 (73.7)	Surgery + AT	1.94	38 (50.0)	OS, PFS	Multivariate	7
Li WT (2015)	China	2009–2013	Up to 2014.12	230	Mean 52	I–IV	224 (97.4)	Radiotherapy or CCRT	2.84	101 (43.9)	OS, PFS	Multivariate	7
Wang Y (2015)	China	1994–2014	Median 51 (3–120)	68	Median 48	I–IIA	NR	Surgery	3.20	37 (54.4)	PFS	Univariate	6
Zhang Y (2014)	China	2005–2008	Up to 2013.6	460	Median 44	I–II	411 (89.3)	Surgery + AT	2.21	230 (50.0)	OS, PFS	Multivariate	7
Wang D (2013)	China	1999–2010	Up to 2011.12	111	Median 42	IB–IIB	98 (88.3)	Surgery + NACT	2.50	52 (46.8)	OS, PFS	Univariate	6
Yee YY (2012)	Korea	1996–2007	Median 52.9 (1–181)	1061	Median 50	IB–IVA	840 (79.2)	Mixed	1.90	575 (54.2)	OS, PFS	Multivariate	8

NR none reported; NLR neutrophil-to-lymphocyte ratio; SCC squamous cell carcinoma; CCRT concurrent chemoradiotherapy; AT adjuvant therapy; NACT neoadjuvant chemotherapy; OS overall survival; PFS progression free survival.

### Impact of NLR on OS

**Figure 2:**
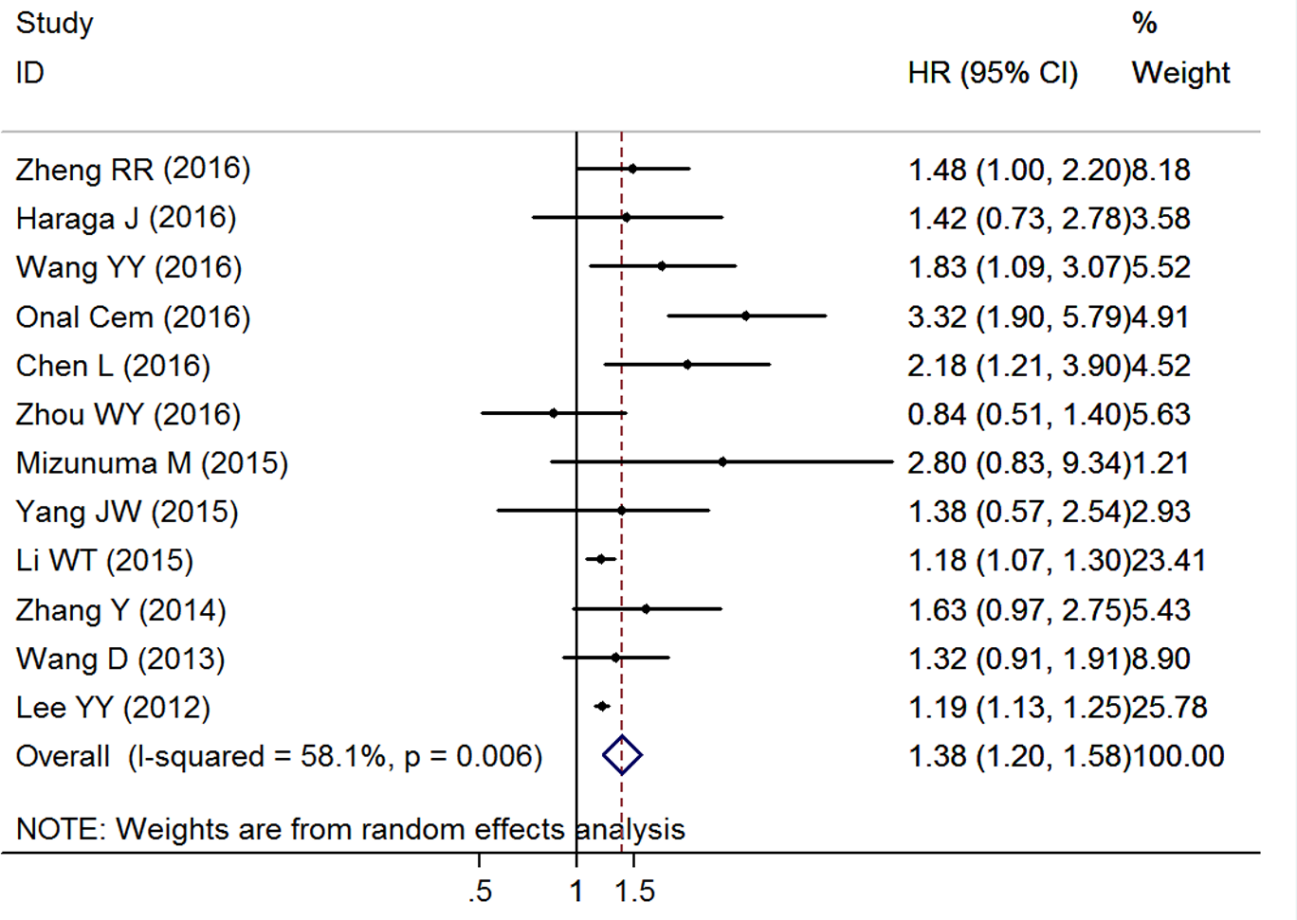
Forest plots of the overall outcome for overall survival Hazard ratios (HRs) for each trial are represented by the squares, and the horizontal lines crossing the square stand for the 95% confidence intervals (CIs). The diamonds represent the estimated pooled effect of the overall outcome for OS in all solid tumors. All *P* values are two-sided.

The combined analysis of 12 studies with 3661 patients showed that patients with elevated NLR were expected to suffer unfavorable OS after treatment (HR = 1.375, 95% CI: 1.200–1.576, *P* < 0.001, random effects; Figure 2). Due to the extreme heterogeneity between studies (*I*^2^ = 58.1%, *P* = 0.006), we conducted subgroup analyses according to the potential confounders, such as study region, clinical stage, sample size, cut-off value, primary treatment, and analysis method. When stratified by clinical stage, elevated NLR predicted poor OS for patients in Stages I–II (HR = 1.388, 95% CI: 1.140–1.691, *P* = 0.001, fixed effects), Stages I–IV (HR = 1.323, 95% CI: 1.112–1.573, *P* = 0.002, random effects) and Stages II–III (HR = 1.829, 95% CI: 1.091-3.065, *P* = 0.022, random effects). Similarly, when grouped based on sample size, the prognostic role of elevated NLR in predicting shorter OS was obvious not only in studies with large sample size (≥ 100) (HR = 1.377, 95% CI: 1.185–1.601, *P* < 0.001, random effects), but also in studies with small sample (< 100) (HR = 1.347, 95% CI: 1.013–1.793, *P* = 0.041, fixed effects). However, subgroup analysis by primary treatment suggested that high NLR had a negative effect on OS both in CC patients receiving surgery with AT (HR = 1. 623, 95% CI: 1.251–2.106, *P* < 0.001, fixed effects), CCRT (HR = 2.092, 95% CI: 1.361–4.382, *P* = 0.003, random effects), radiotherapy or CCRT (HR = 1.186, 95% CI: 1.074–1.309, *P* = 0.001, fixed effects), and mixed treatments (HR = 1.190, 95% CI: 1.130–1.250, *P* < 0.001, random effects), but not in patients receiving surgery with NACT (HR = 1. 127, 95% CI: 0.834–1.423, *P* = 0.436, fixed effects). Moreover, the significant association of elevated NLR and worse OS did not change regardless of the subgroup analyses of study region, cut-off value, and analysis method (Table 2).

**Table 2:** Summary of the meta-analysis results

Analysis	Ctegories	*n*	Model	HR (95% CI)	*Z*	*P*	Heterogeneity	
							*I*^2^	*P*_h_
**Overall survival (OS)**		**12 (3661)**	**Random**	**1.375 (1.200–1.576)**	**4.58**	**< 0.001**	**58.1%**	**0.006**
Study region:	Eastern countries	11 (3426)	Fixed	1.203 (1.141–1.256)	8.31	< 0.001	25.9%	0.197
	Western countries	1 (235)	random	3.322 (1.905–5.792)	4.23	< 0.001	NA	NA
Clinical stage:	Stage I–II	6 (1924)	Fixed	1.388 (1.140–1.691)	3.26	0.001	23.0%	0.261
	Stage I–IV	5 (1677)	Random	1.323 (1.112–1.573)	3.16	0.002	73.8%	0.004
	Stage II–III	1 (60)	Random	1.829 (1.091–3.065)	2.29	0.022	NA	NA
Sample size:	≥ 100	7 (3299)	Random	1.377 (1.185–1.601)	4.17	< 0.001	69.5%	0.003
	< 100	5 (362)	Fixed	1.347 (1.013–1.793)	2.05	0.041	33.6%	0.198
Cut-off value:	≥ 2.5	6 (1522)	Random	1.568 (1.161–2.118)	2.93	0.003	68.4%	0.007
	< 2.5	6 (2139)	Random	1.369 (1.071–1.749)	2.51	0.012	49.8%	0.077
Primary treatment:	Surgery + NACT	2 (186)	Fixed	1.127 (0.834–1.523)	0.78	0.436	47.6%	0.167
	Surgery + AT	4 (1738)	Fixed	1.623 (1.251–2.106)	3.64	< 0.001	0.0%	0.606
	CCRT	3 (390)	Random	2.092 (1.361–4.382)	3.00	0.003	52.2%	0.124
	Radiotherapy or CCRT	2 (286)	Fixed	1.186 (1.074–1.309)	3.38	0.001	48.7%	0.163
	Mixed	1 (1061)	Random	1.190 (1.130–1.250)	6.76	< 0.001	NA	NA
Analysis method:	Multivariate	8 (2600)	Random	1.354 (1.145–1.600)	3.55	< 0.001	68.6%	0.002
	Univariate	4 (1061)	Fixed	1.470 (1.172–1.843)	3.33	0.001	0.0%	0.793
**Progression free survival (PFS)**		**10 (2452)**	**Random**	**1.646 (1.313–2.065)**	**4.31**	**< 0.001**	**80.3%**	**< 0.001**
Study region:	Eastern countries	9 (2217)	Random	1.496 (1.227–1.823)	3.98	< 0.001	71.9%	< 0.001
	Western countries	1 (235)	Random	3.579 (2.106–6.082)	4.71	< 0.001	NA	NA
Clinical stage:	Stage I–II	4 (715)	Fixed	1.740 (1.375–2.202)	4.61	< 0.001	38.0%	0.184
	Stage I–IV	5 (1677)	Random	1.460 (1.097–1.943)	2.60	0.009	83.3%	< 0.001
	Stage II–III	1 (60)	Random	2.135 (1.274–3.579)	2.88	0.004	NA	NA
Sample size	≥ 100	5 (2097)	Random	1.469 (1.096–1.970)	2.57	0.010	81.8%	< 0.001
	< 100	5 (355)	Fixed	1.752 (1.452–2.113)	5.85	< 0.001	17.1%	0.306
Cut-off value	≥ 2.5	6 (795)	Random	1.606 (1.193–2.161)	3.12	0.002	71.8%	0.003
	< 2.5	4 (1657)	Random	1.804 (1.100–2.958)	2.34	0.019	83.9%	< 0.001
Primary treatment:	Surgery	4 (715)	Fixed	1.740 (1.375–2.202)	4.61	< 0.001	38.0%	0.184
	CCRT	3 (390)	Fixed	2.457 (1.762–3.428)	5.30	< 0.001	45.6%	0.159
	Radiotherapy or CCRT	2 (286)	Random	1.282 (1.074–1.531)	2.75	0.006	73.3%	0.053
	Mixed	1 (1061)	Random	1.130 (1.081–1.180)	5.41	< 0.001	NA	NA
Analysis method:	Multivariate	6 (2118)	Random	1.650 (1.226–2.220)	3.30	0.001	86.1%	< 0.001
	Univariate	4 (334)	Fixed	1.635 (1.283–2.083)	3.98	< 0.001	0.0%	0.537

NACT neoadjuvant chemotherapy; AT adjuvant therapy; CCRT concurrent chemoradiotherapy.

*P* denotes *P* value for statistical significance based on *Z* test; *P*_h_ denotes *P* value for heterogeneity based on *Q* test. HR hazard ratio; CI confidence interval; NA not available.

### Impact of NLR on PFS

**Figure 3:**
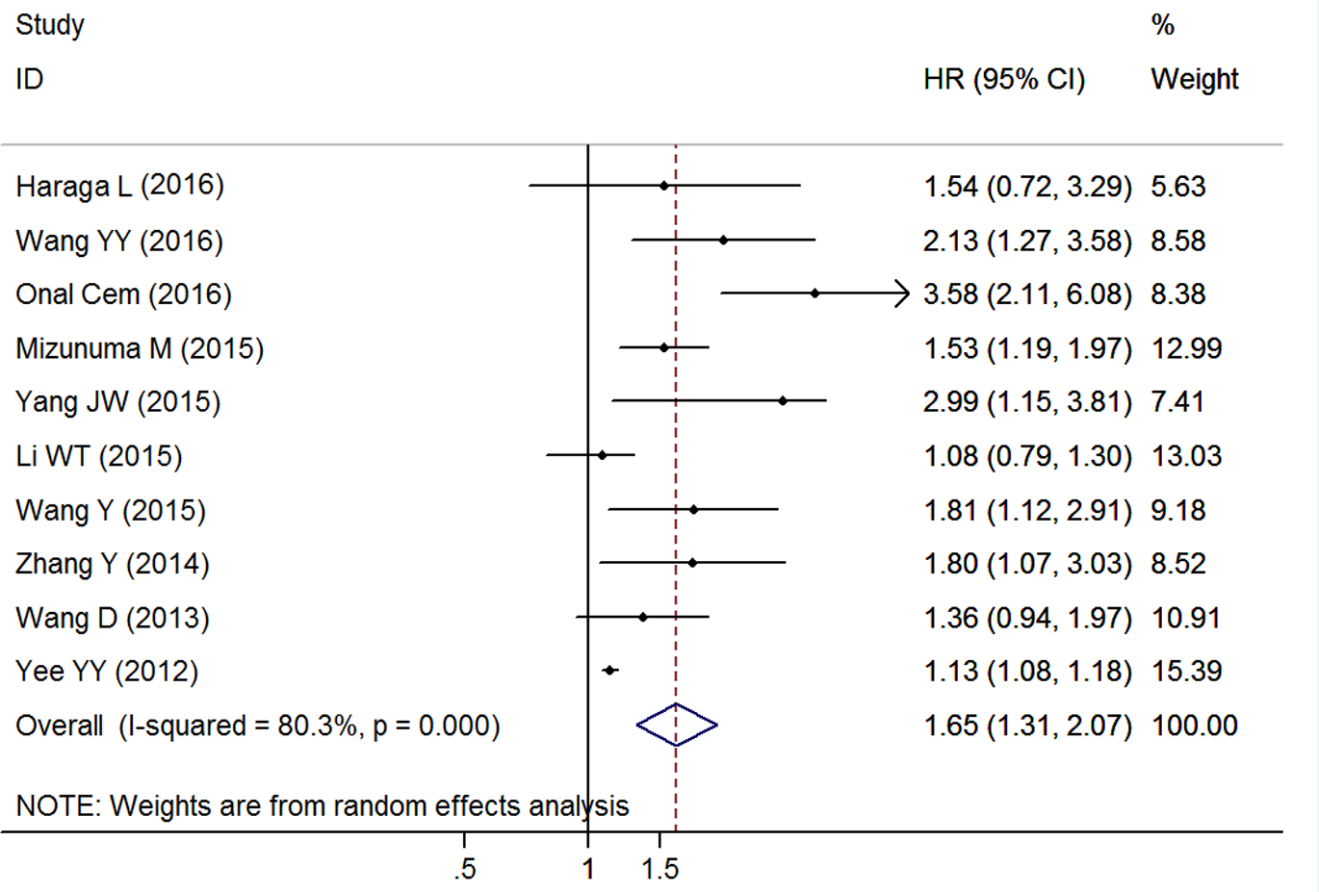
Forest plots of the overall outcome for progression-free survival Hazard ratios (HRs) for each trial are represented by the squares, and the horizontal lines crossing the square stand for the 95% confidence intervals (CIs). The diamonds represent the estimated pooled effect of the overall outcome for PFS in all solid tumors. All *P* values are two-sided.

Ten researches with 2452 cases represented the data of pretreatment NLR and PFS in patients with CC. The pooled result showed that increased NLR was significant correlated with worse PFS (HR = 1.646, 95% CI: 1.313–2.065, *P* < 0.001, random effects; Figure 3) with extreme heterogeneity (*I*^2^ = 80.3%, *P* < 0.001). Stratification by primary treatment, the obvious relationship of elevated NLR and poor PFS was found in patients receiving surgery no matter with NACT, AT, or alone (HR = 1.740, 95% CI: 1.375–2.202, *P* < 0.001, fixed effects), CCRT (HR = 2. 457, 95% CI: 1.762-3.428, *P* < 0.001, fixed effects), radiotherapy or CCRT (HR = 1.282, 95% CI: 1.074–1.531, *P* = 0.006, random effects), and mixed treatments (HR = 1.130, 95% CI: 1.081–1.180, *P* < 0.001, random effects). Similarly, this trend was also observed with the stratification of clinical stage, such as Stages I–II (HR = 1.740, 95% CI: 1.375–2.202, *P* < 0.001, fixed effects), Stages I–IV (HR = 1.460, 95% CI: 1.097-1.943, *P* = 0.009, random effects) and Stages II–III (HR = 2.135, 95% CI: 1.274–3.579, *P* = 0.004, random effects). In addition, when the included cohorts were stratified by study region, sample size, cut-off value, and analysis method, the results did not show any significant change (Table 2).

### Association between NLR and clinicopathologic parameters

There were 6 trials with 1151 cases reported the correlation between NLR and tumor size, and the pooled outcome indicated that high NLR was related to larger tumor size (odds ratio [OR] = 1.780, 95% CI: 1.090–2.908, *P* = 0.021, random effects). The relationship of NLR and clinical stage was reported in 4 studies, and a significant association was found between elevated NLR and advanced clinical stage (OR = 2.443, 95% CI: 1.730–3.451, *P* < 0.001, fixed effects). Six studies reported the connection of NLR and lymph node metastasis, and the conjoined result declared that high NLR was related to positive lymph node metastasis (OR = 2.380, 95% CI: 1.775–3.190, *P* < 0.001, fixed effects). However, NLR was not significantly associated with histologic grade (OR = 1.317, 95% CI: 0.968–1.792, *P* = 0.080, fixed effects) and histologic type (OR = 1.007, 95% CI: 0.792–1.281, *P* = 0.955, fixed effects) (Table 3).

**Table 3:** Summary of the association of NLR and clinopathological parameters in cervical cancer

Category	*n*	Model	OR (95% CI)	*Z*	*P*	Heterogeneity	
						*I*^2^	*P*_h_
Histologic grade (poor vs. well or moderate)	5 (916)	Random	1.317 (0.968–.792)	1.75	0.080	54.0%	0.069
Tumor size (≥ 4 cm vs. < 4 cm)	6 (1151)	Random	1.780 (1.090–2.908)	2.30	0.021	66.3%	0.011
Clinical stage (III or IV vs.I or II)	4 (1437)	Fixed	2.443 (1.730–3.451)	5.07	< 0.001	0.0%	0.768
Lymph node metastasis (positvie vs. negative)	6 (998)	Fixed	2.380 (1.775–3.190)	5.79	< 0.001	0.0%	0.515
Histologic type (SCC vs. non SCC)	6 (2173)	Fixed	1.007 (0.792–1.281)	0.06	0.955	0.0%	0.421

*P* denotes *P* value for statistical significance based on *Z* test; *P*_h_ denotes *P* value for heterogeneity based on *Q* test. OR odds ratio; CI confidence interval; SCC squamous cell carcinoma.

### Sensitivity analysis and meta-regression analysis

**Figure 4:**
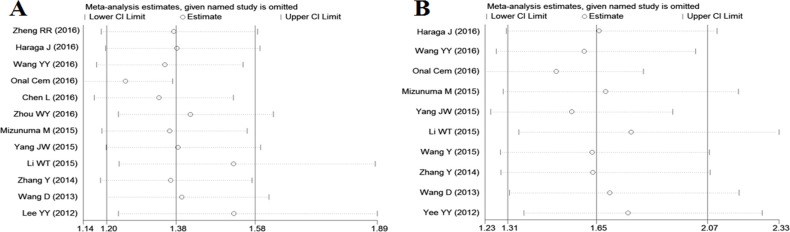
Effect of individual studies on pooled hazard ratios (HR) for the relationship between neutrophil-to-lymphocyte ratio and prognosis of cervcal cancer (**A**) Sensitivity analysis for overall survival. The vertical axis at 1.38 indicates the overall HR, and the two vertical axes at 1.20 and 1.58 indicate its 95% confidence interval (CI). Every hollow round indicates the pooled HR when the left study was omitted in a meta-analysis with a random model. The two ends of every broken line represent the respective 95% CI. (**B**) Sensitivity analysis for progression-free survival. The vertical axis at 1.65 indicates the overall HR, and the two vertical axes at 1.31 and 2.07 indicate its 95% CI. Every hollow round indicates the pooled HR when the left study was omitted in a meta-analysis with a5 random model. The two ends of every broken line represent the respective 95% CI.

Sensitivity analysis suggested that no point estimate of the omitted individual dataset lay outside the 95% CI of the combined analysis based on the overall HR estimate of OS and PFS (Figure 4).

We conducted meta-regression analysis to investigate the potential source of heterogeneity among studies for OS and PFS. In multivariate analysis, the results showed that study region (*P* = 0.143), clinical stage (*P* = 0.815), sample size (*P* = 0.784), cutoff value (*P* = 0.726), primary treatment (*P* = 0.870) and analysis method (*P* = 0.707) did not contribute to the source of heterogeneity for OS. Moreover, the data demonstrated that study region (*P* = 0.053), clinical stage (*P* = 0.852), sample size (*P* = 0.092), cutoff value (*P* = 0.129), primary treatment (*P* = 0.146) and analysis method (*P* = 0.525) did not account for the source of heterogeneity for PFS.

### Publication bias

**Figure 5:**
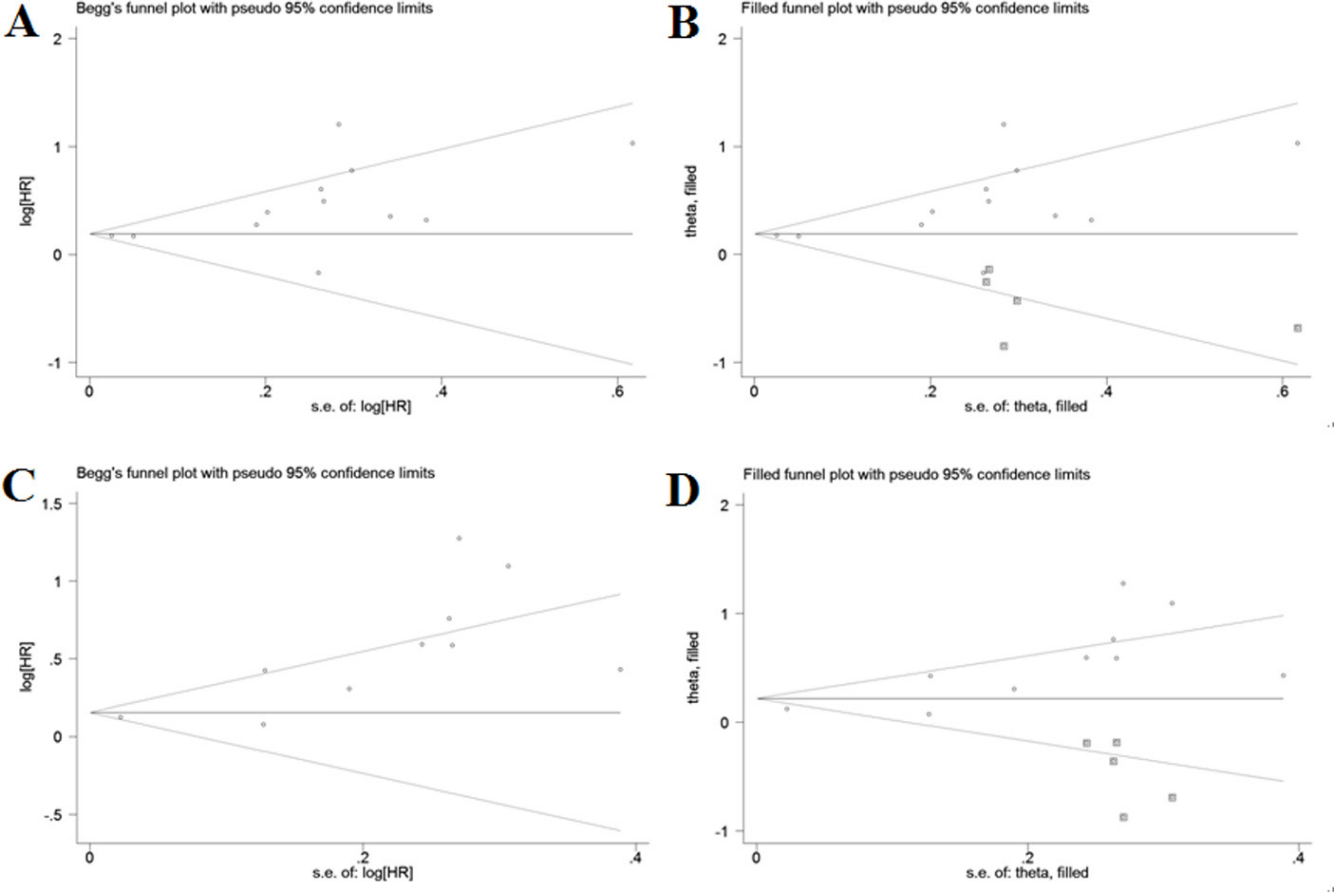
Funnel plots for assessment of potential publication bias in studies of neutrophil-to-lymphocyte ratio in patients with cervical cancer Each study represented by one circle. The horizontal line represented the pooled effect estimate. (**A**) Funnel plot of publication bias for studies reporting overall survival. (**B**) Funnel plot adjusted with trim and fill method for studies reporting overall survival. (**C**) Funnel plot of publication bias for studies reporting progression-free survival. (**D**) Funnel plot adjusted with trim and fill method for studies reporting progression-free survival.

Although there was no publication bias by Begg's test (*P* = 0.115), a significant publication bias was found by Egger's test (*P* = 0.021) for OS, and the funnel plot showed a certain degree of apparent asymmetry (Figure 5A), which indicated potential publication bias. The trim-and-fill analysis showed that five non-published studies were needed to balance the funnel plot (Figure 5B). The adjusted HR and 95% CI were attenuated but remains significant (pooled HR = 1.208; 95% CI = 1.042–1.401; *P* = 0.012; random effects), thereby suggesting that the potential publication bias had minimal impact on the overall outcome. Similarly, with regard to PFS, a significant publication bias was observed by Egger's test (*P* = 0.003) but not by Begg's test (*P* = 0.107), which was also confirmed by the funnel-plot shape (Figure 5C). After adjusted by the trim-and-fill analysis, five more studies were added into the funnel plot (Figure 5D) and the recalculated results did not changed significantly (pooled HR = 1.245; 95% CI = 1.013–1.530; *P* = 0.038; random effects), indicating the robustness of the results. Moreover, reports assessing the relationship between NLR and histologic grade (Begg's test: *P* = 0.086; Egger's test: *P* = 0.095), tumor size (Begg's test: *P* = 0.452; Egger's test: *P* = 0.363), clinical stage (Begg's test: *P* = 0.734; Egger's test: *P* = 0.959), lymph node metastasis (Begg's test: *P* = 0.060; Egger's test: *P* = 0.089), as well as histologic type (Begg's test: *P* = 0.452; Egger's test: *P* = 0.469) also showed no publication bias.

## DISCUSSION

NLR has been frequently used as an inflammatory marker, while its prognostic value in various tumors was reported during the last decade. The impact of high NLR on shorter survival time after curative intent resection of solid tumors has been declared in a recent meta-analysis [38], but the included studies did not concern cervical cancer, so the prognostic role of NLR on CC was still uncertain. We conducted this first meta-analysis to examine the association between NLR and prognosis as well as clincopathological parameters in CC. The combined outcomes of 3729 patients from 13 studies demonstrated that elevated pretreatment NLR predicted poor OS and PFS in CC patients, regardless of the patients’ clinical stage. Subgroup by primary treatment showed that elevated NLR had not a prognostic significance concerning OS in patients receiving surgery with NACT, however, more studies were needed to consolidate or overthrow the conclusion since only two studies with 186 patients were conducted based on this primary treatment. Though with heterogeneity, the prognostic significance is not weakened by subgroup analyses. Therefore, NLR is a promising prognostic marker helpful for the clinical decision-making process regarding CC treatment and outcomes.

Although sensitivity analysis suggested no individual study dominated the meta-analysis results, the results of our meta-analysis should be interpreted cautiously because the heterogeneity of the OS and the PFS estimations were extreme, even when we conducted subgroup analyses. Meta-regression analysis was performed using variables such as study region, clinical stage, sample size, cutoff value, primary treatment, and analysis method, to further investigate the source of heterogeneity; however, none of the these confounders could completely explain the heterogeneity. Moreover, despite the broad search criteria, a significant publication bias remained possible among the studies concerning OS and PFS (*P* < 0.05), which may have inflated the overall results. A trim-and-fill analysis was performed to recalculate the pooled results, and the adjusted HRs and 95% CIs reinforced the prognostic role of NLR in both OS and PFS but remained statistically. This finding indicated that the publication bias may not have a systematic influence on the pooled findings.

Synthesized data of the relationship between the pretreatment NLR and clinicopathological features suggested that increased NLR was significantly associated with larger tumor size, advanced clinical stage, and positive lymph node metastasis. All of these factors have been documented to be the powerful variables related to CC progression and compromise long-term survival [39–42]. Herein, high NLR is closely associated with more aggressive tumor behavior which is contributed to shorter OS and PFS. Therefore, CC patients with large tumor size, advanced clinical stage, or positive lymph node metastasis would benefit most from NLR evaluation to make clinical decisions.

Although several inflammatory markers, such as CRP and mGPS, have been reported to show prognostic value in CC, they are not the routine pre-treatment assessments in grass-roots hospitals, especially those in economic less-developed areas. Due to the need of high cost and advanced technology, protein biomarkers and gene polymorphism also can not be used as routine prognostic predictor in general clinical application. By contrary, haematological test is carried out routinely at a low cost before the treatment of cancer patients, making it a convenient and reproducible laboratory parameter to reflect the inflammatory status in body. Moreover, it is well known that systematic inflammatory response represents as alterations in peripheral white blood cells (WBC) subset populations, particularly neutrophilia with a relative lymphocytopaenia [43]. Both pretreatment neutrophil count and lymphocyte count can be the independent prognostic factors of CC [44–46], but their numerical values are not stable when they solely act as single inflammatory index, so it is more appropriate to combine them for an independent indicator. The results of our meta-analysis suggest that NLR provides independent prognostic information, and is encouraged to be routinely monitored to predict clinical outcome of CC patients, regardless of the therapeutic intervention and tumor stage.

According to the study of Gwak et al. [47], NLR and WBC subset populations was higher in female patients than in male patients after gastrectomy due to gastric cancer, which indicated that females may be more vulnerable to changes in immune response following malignant diseases or surgical stress. Thus, inflammatory markers may be with greater diagnostic and prognostic efficacies for malignances in female patients. Unlike inflammation in wound healing functions as destroying the infectious agents, inflammation during cancer fails to undergo healing process and persists to acquire a chronic condition leading to a persistent infection [48]. There is evidence that human papillomavirus (HPV) infection is necessary for cervical carcinogenesis, but it is not sufficient for the development of the neoplasia [49]. While many HPV infections are transient, women with co-infections are at increased risk of persistent HPV infections compared with uninfected women, which is regarded as a critical event for cervical cancer development. Chlamydia trachomatis acts in a synergistic manner by producing a local immune perturbation that decreases the number of antigen-presenting cells involved in HPV clearance and facilitates HPV cellular transformation, resulting in a viral persistence during carcinogenesis [50]. Also, simultaneous infection with human immunodeficiency virus (HIV) and multiple concurrent HPVs contributes to elevated cervical inflammation and a greater risk of developing precancerous lesions more than either condition on its own [51]. Moreover, the co-infections can form an inflammatory microenvironment to stimulate HPV cell entry, replication and viral integration by increasing the release of oxidative stress proteins that can enhance cellular DNA breaks [52]. In addition, an increasing body of evidence suggests that inflammation mediates different steps of tumorigenesis through the infiltration of white blood cells, especially tumor-associated macrophages (TAMs); the presence of cytokines, such as TNF, IL-1, and IL-6; the secretion of chemokines, such as CCL2 and CXCL8; as well as the occurrence of tissue remodeling and angiogenesis [53, 54]. Therefore, paying more attention to co-infection may help to better understand the impact of inflammation on tumor progression, and is important for developing new therapeutic strategies based on the nature of malignancy.

At present, the specific mechanism involved in the interaction between elevated NLR and poor prognosis of CC was incompletely understood. Here are some possible explanations that can be used for interpreting this result. First, lymphocytes play an important role in the antitumor immunological reaction by preventing the proliferation and metastasis of malignant cells [55]. The subtypes of lymphocytes, such as CD3^+^ T cells, CD8^+^ T cells, Th1 CD4^+^ T cells, and p46^+^ natural killer cells, are essential to the antitumor immunological reaction and have been proved to improve the survival of patients with malignancy [56]. However, systemic inflammation response from malignant cells could cause immune suppression, by which tumor cells can escape host immune surveillance. An important sign of immune escape is T-lymphocyte dysfunction. T-lymphocytes are a common kind of tumor infiltrating lymphocytes (TILs). Patients with elevated levels of TILs infiltration surrounding the primary tumor site have a good prognosis than those with less or no infiltration [57]. It has also been reported that tumor cells can inhibit cytotoxic T lymphocyte infiltration by producing immunosuppressive cytokines, such as vascular endothelial growth factor (VEGF), transforming growth factor-β (TGF-β), or IL-10, and by reducing IL-2, a cytokine that can maintain cytotoxic T lymphocyte function [58]. A low lymphocyte count has been found in many human neoplasms, and it is often associated with worse clinical outcomes, which may be attributed to the depressed lymphocyte-mediated immune response to the tumor [59]. Second, neutrophils account for about 60% of all leukocytes in the bloodstream, and are considered to be the first line of defense during inflammation and infection [60]. It has become clear that infiltrating neutrophils were found in many types of tumor tissue, and the tumor-associated neutrophils (TANs) are related to advanced disease for cancer patients. TANs have been showed to be the primary source of circulating vascular endothelial growth factor (VEGF), which plays a critical role in accelerating tumor-related angiogenesis [61]. TANs can induce a chronic inflammation and then create an immunosuppressive state, which inhibits the cytolytic ability of T lymphocytes, by secreting cytokines (IL-1β, TNF-α, IL-6, and IL-12) and arginase 1. Serine proteases, such as elastase and cathepsin G, secreted by TANs, can degrade the basement membrane and promote tumor cell invasion through the basement membrane [62]. Once in circulation, neutrophils also have a direct effect on helping tumor cells to survive by inducing proliferation. Moreover, certain tumors induce neutrophils to produce some special cytokines, such as Oncostatin and hepatocyte growth factor (HGF), to activate tumor cells to become more invasive [63]. Therefore, elevated NLR, usually caused by an increased number of neutrophils and/or a decrease in lymphocytes count, denotes that the balance of pro-tumor and anti-tumor status has been broken and is skewed to a pro-tumor inflammatory condition, generating a favorable immune microenvironment for tumor progression and leads to a worse prognosis.

It should be acknowledged that there are still some defects in our meta-analysis. First, the cut-off values for defining elevated NLR in each individual study were not inconsistent because no acknowledged threshold was available, which may have contributed to heterogeneity to some extent. Second, the therapeutic approach and the follow-up period of the individual studies were not unified, and these differences may also be a potential source of heterogeneity. Third, some studies only provided the HRs and their 95% CIs calculated from Kaplan-Meier curves or univariate analysis rather than from multivariate analysis, which may slightly different from the actual HRs, and impair the accuracy of the pooled estimates. Fourth, our meta-analysis was limited to the published literature, and information from studies with negative outcomes and small sample sizes would unavoidably be missed, because positive results with large populations are more inclined to being published. Finally, this meta-analysis was at study level, and confounding variables at the patient level were not incorporated into the analysis.

In conclusions, our meta-analysis demonstrated that pretreatment elevated NLR is closely associated with poor survival outcome and unfavorable clinicopathological parameters in CC. We conclude that NLR can serve as a convenient, inexpensive, simple, and reproducible index to identify patients who may suffer high risk of poor prognosis and benefit less from the antitumor therapies, which is helpful to facilitate the management strategy accordingly. However, due to the limitations of this meta-analysis, further large prospective studies are needed to better understand the prognostic value of NLR in CC.

## MATERIALS AND METHODS

### Search strategy

A comprehensive literature search was conducted through PubMed (Medline), Embase, the Cochrane Library, Web of Science databases, and China National Knowledge Infrastructure databases. The search was updated to December 15, 2016 based on the following terms: “NLR or neutrophil to lymphocyte ratio or neutrophil-to-lymphocyte ratio or neutrophil lymphocyte ratio” and “prognosis or survival or outcome or mortality” and “cervical cancer or cervical carcinoma or cervix cancer or cervix carcinoma”. The citation lists of the included studies were also screened to find more relevant studies. This meta-analysis was performed according to the guideline of Preferred Reporting Items for Systematic Reviews and Meta-analysis [64].

### Inclusion and exclusion criteria

All candidate studies were independently reviewed by two reviewers (W.J.Y. and C.M.Y.). Discrepancies were resolved by discussion. Publications were regarded as eligible when they satisfy all of the following criteria: (1) patients with cervical cancer in the studies were confirmed histopathologically; (2) reported the association between NLR and the survival outcome of OS or PFS; (3) the NLR was evaluated before any treatment; (4) HRs and their 95% CIs were provided in the original data or extracted from the sufficient information; (5) to be published as full texts in any language. Articles were excluded if they met any of the following characteristics: (1) abstracts, letters, reviews, editorials, case reports, expert opinions, nonclinical studies or nonhuman researches; (2) insufficient data to evaluate the HRs and 95% CIs; (3) overlapping or duplicate data; (4) sample size less than 50.

### Data extraction and quality assessment

Data was extracted from the studies including: (1) the first author's name, publication year, study region, duration time, follow-up months, sample size, quality scores; (2) clinical features including clinical stage, tumor histopathology, primary treatment; (3) cut-off value used to define “high NLR”; (4) survival outcomes including OS and PFS; (5) HR estimation. If both HRs and the corresponding 95% CIs of univariate and multivariate analyses were provided in the study, only the latter was applied to data synthesis because it is more precise and it considers confounding factors. In the absence of results from multivariate analysis, HR was extracted from univariate analysis or calculated using Kaplan–Meier survival curves [65]. If all the patients in the individual study were treated with operative therapy and only some of them received additional nonsurgical therapy in the follow up, including chemotherapy and/or radiation therapy, the study was classified into surgical subgroup. By contrary, if all the patients in the individual study received non-surgical intervention followed by only some of the patients undergoing surgery, the study was classified into nonsurgical subgroup.

The quality of included studies was evaluated by Newcastle–Ottawa Scale (NOS) according to the following categories: selection, comparability, and outcome of interest [66]. The total score of NOS ranged from 0 to 9, and we considered studies as high quality if they met at least six scores.

### Statistical analysis

The combined HR and 95% CI were used to assess the strength of NLR with survival endpoints (OS, and PFS) based on data extracted from the eligible studies. HR > 1 with 95% CI exceeding 1 indicated an increased hazard of poor prognosis for patients with elevated NLR. The statistical significance of the pooled HR was determined by a *Z*–test. The results are considered statically significant if *P* < 0.05. Subgroup analyses were conducted according to study region, clinical stage, sample size, cut-off value, primary treatment, and analysis method. Meta-regression analysis was also performed to determine the potential sources of heterogeneity. For the pooled analysis of correlation between NLR and clinicopathological features (histologic grade, tumor size, clinical stage, lymph node metastasis, and histologic type), ORs and the corresponding 95% CI were combined to estimate the effect. STATA version 11.0 (STATA Corporation, College Station, TX, USA) was used for all statistical analysis. All statistical tests were two sided.

Heterogeneity assumption was examined by the Cochran's Q stastic and Higgins *I*^2^ metric. *P* < 0.10 or *I*^2^ > 50% was considered as a measurement of extreme heterogeneity [67]. A random-effects model (DerSimonian and Laird method) was performed to calculate the pooled HR estimation of each study when extreme heterogeneity existed. Otherwise, a fixed-effect model was used (Mantel-Haenszel method) [68]. Sensitivity analysis was conducted by sequential omitting each individual study to validate the stability of the meta-analysis outcomes. Potential publication bias was evaluated by Begg's and Egger's Asymmertry tests quantitatively [69], and by funnel plots visually. If significant publication bias existed, trim and fill method was performed to validate the robust of the meta-analysis results [70]. A two-tailed *P value* of less than 0.05 was defined as statistically significance.
